# Extracellular Vesicles Derived From Olfactory Ensheathing Cells Promote Peripheral Nerve Regeneration in Rats

**DOI:** 10.3389/fncel.2019.00548

**Published:** 2019-12-06

**Authors:** Bing Xia, Jianbo Gao, Shengyou Li, Liangliang Huang, Teng Ma, Laihe Zhao, Yujie Yang, Jinghui Huang, Zhuojing Luo

**Affiliations:** ^1^Department of Orthopaedics, Xijing Hospital, Fourth Military Medical University, Xi’an, China; ^2^Department of Orthopaedics, The General Hospital of Central Theater Command of People’s Liberation Army, Wuhan, China

**Keywords:** olfactory ensheathing cells, extracellular vesicles, peripheral nerve injury, nerve regeneration, functional recovery

## Abstract

Accumulating evidence showed that extracellular vesicles (EVs) and their cargoes are important information mediators in the nervous system and have been proposed to play an important role in regulating regeneration. Moreover, many studies reported that olfactory ensheathing cells (OECs) conditioned medium is capable of promoting nerve regeneration and functional recovery. However, the role of EVs derived from OECs in axonal regeneration has not been clear. Thereby, the present study was designed to firstly isolate EVs from OECs culture supernatants, and then investigated their role in enhancing axonal regeneration after sciatic nerve injury. *In vitro* studies showed that OECs-EVs promoted axonal growth of dorsal root ganglion (DRG), which is dose-dependent and relies on their integrity. *In vivo* studies further demonstrated that nerve conduit containing OECs-EVs significantly enhanced axonal regeneration, myelination of regenerated axons and neurologically functional recovery in rats with sciatic nerve injury. In conclusion, our results, for the first time, demonstrated that OECs-EVs are capable of promoting nerve regeneration and functional recovery after peripheral nerve injuries in rats.

## Introduction

Olfactory ensheathing cells (OECs) are specialized glial cells located in the olfactory system which guide growing axons of olfactory receptor neurons of the olfactory mucosa through the cribriform plate and travel to the olfactory bulb, thereby allowing axons to travel from the peripheral nervous system (PNS) to the central nervous system (CNS; Radtke and Kocsis, 2014). Neurogenesis allows for the replacement of olfactory receptor neurons, which is ongoing throughout the whole life in mammals (Mackay-Sim and Kittel, 1991). Many previous studies have shown that OECs are capable of enhancing nerve regeneration in both PNS and CNS (Verdú et al., 1999; Franssen et al., 2007; Radtke et al., 2009). However, the mechanism for the beneficial effect of OECs on axonal regeneration has not been clear thus far.

It has been recognized that functions of the nervous system are critically dependent on proper intercellular communication. It has been reported that long-term cultured OECs failed to support regeneration and neural repair (Verdú et al., 1999; Radtke et al., 2010), which (Verdú et al., 1999) might be attributable to the decreased viability of the transplanted OECs due to the unsuitable microenvironment. Further studies have shown that loss or change of some specific classes of cell surface receptors in long-term cultured OECs might be partially responsible for their poor performance in promoting axonal regeneration (Qi et al., 2013). The communications between OECs and neural cells can be localized or distant. The local communication has been extensively recognized, such as adhesion molecules, gap junctions, and synapses (Mittelbrunn and Sánchez-Madrid, 2012). In contrast, little information has been available for distant communication, which is important for the role of OECs in the nervous system since studies have shown that OECs-conditioned medium is capable of promoting nerve regeneration (Gu et al., 2017).

Increasing evidence has revealed that extracellular vesicles (EVs) are emerging as a novel and promising form of information exchange among cells (Korkut et al., 2013; Janas et al., 2016). EVs carry different types of cargoes and interact with target cells to regulate their functions (Mittelbrunn and Sánchez-Madrid, 2012; Borroto-Escuela et al., 2015) and thus act as messengers or information mediators (Bátiz et al., 2015). It has been recognized that EVs contain complex cargoes including proteins, lipids and various RNA species, which vary depending on the origin of resource cells and their physiological and pathological conditions (Colombo et al., 2014; Abels and Breakefield, 2016). Many studies have demonstrated that EVs play an important role in regulating nerve regeneration. EVs derived from Schwann cells (SCs), mesenchymal stem cells (MSCs), and macrophages have been found to be capable of enhancing peripheral nerve regeneration (Pegtel et al., 2014; Zhang et al., 2015). In addition, EVs has been isolated from astrocytes (Frühbeis et al., 2012), microglia (Potolicchio et al., 2005), and oligodendrocytes (Krämer-Albers et al., 2007), serving as modulators of cell-to-cell communication in the healthy and diseased brain. However, little information is available for the isolation of EVs from OECs thus far. In addition, there has been no relevant research on OECs-EVs in peripheral nerve repair. Therefore, the present study was designed to isolate EVs from OECs and identify their role in peripheral nerve regeneration.

In this study, we first isolated EVs by serial ultracentrifugation from OECs culture supernatants and characterized them by Western blotting analysis, transmission electron microscopy (TEM) and nanoparticle tracking analysis (NTA). The OECs-EVs were then labeled with PKH26 and added to dorsal root ganglion (DRG) neurons to investigate the interactions between OECs-EVs and neurons. In addition, the effect of OECs-EVs on neurite outgrowth was examined in purified DRG neurons and explant cultures. Finally, the effect of OECs-EVs on nerve regeneration and functional recovery was studied in rats with 5-mm sciatic nerve defects.

## Materials and Methods

### Isolation and Characterization of OECs Cultures

OECs were harvested and purified from the olfactory bulbs of Sprague–Dawley (SD) male rats weighing 220 g following previously established protocol with some modifications (Nash et al., 2002; Goulart et al., 2016). All procedures were conducted under a protocol in accordance with the Guide for the Care and Use of Laboratory Animals (National Institutes of Health Publication No 85-23, revised 1985) and approved by the Animal Research Committee of The Fourth Military Medical University, People’s Republic of China. Briefly, the SD rat (provided by the Experimental Animal Center of the Fourth Military Medical University) was killed by isoflurane. The head was immersed in the 0.5% iodine solution for 3 min and deiodinated with 75% alcohol. The skull was opened and the olfactory bulbs were dissected and the caudal one-third of the bulb were sliced and discarded with as much white matter as possible to obtain the outer nerve layer. The obtained tissue was finely minced with scalpel blades on culture dishes and dissociated with 0.25% trypsin/0.02% EDTA (Sigma–Aldrich, St. Louis, MO, USA) digestion solution for 20 min at 37°C on a rotary shaker in a CO_2_ incubator. The digestion was quenched by adding Dulbecco’s Modified Eagle Medium/Nutrient Mixture F-12 (DMEM/F-12; Gibco, Grand Island, NY, USA) containing 10% fetal bovine serum (FBS; Gibco). The cell’s suspension was then centrifuged at 350 *g* for 5 min, the supernatant was discarded, and the cell pellet was gently triturated with fire-polished pasture pipettes. The cell suspension was resuspended in DMEM/F-12 supplemented with 10% FBS and 1% antibiotics (penicillin and streptomycin solution), preplaced for 18 h in a culture flask at 37°C in a 5% CO_2_ incubator in preparation for fibroblast removal. Next, non-adherent cells in cell suspension were gently transferred to a second uncoated culture flask for astrocyte removal, and incubated under the same conditions for 36 h. Finally, the OECs in the cell suspension were adhered onto a precoated poly-L-lysine (10 mg/ml; Sigma–Aldrich) coated culture plate in DMEM/F-12 complete medium containing 10% FBS, 1% antibiotics (penicillin and streptomycin solution), basic fibroblast growth factor (bFGF, 0.01 mg/L; Sigma–Aldrich) and 2 μM forskolin (Sigma–Aldrich). OECs were maintained in an incubator at 37°C, 5% CO_2_, and the culture medium was refreshed every 2–3 days. Once confluency was reached, OECs were detached using trypsin and used in the remaining experiments.

Colocalization of p75 Neurotrophin receptor and smooth muscle α-actin (SMA; or calponin) can be used together as definitive markers for OECs both *in vitro* and *in vivo* (Jahed et al., 2007). The purity of the OECs cultures was determined by co-staining of OECs with p75^NTR^ (ab52987; Abcam, Cambridge, UK) and SMA (A2547; Sigma–Aldrich) antibody. The cell nucleus was stained with 4′,6-diamidino-2-phenylindole (DAPI) solution (Sigma–Aldrich) and numbers of p75^NTR^ and SMA positive cells and DAPI-labeled cells were counted. The final preparations consisted of a high purity (>96%) of OECs which was confirmed by images acquired from a fluorescence confocal microscope (A1+, Nikon, Japan).

### Extracellular Vesicles Isolation and Characterization

Passage two primary OECs were employed to generate EVs. Briefly, 2 × 10^6^ cells were plated in a pre-coated T75 flask and were maintained in DMEM/F-12 complete medium. When cells were confluent (90–100%), they were washed twice with PBS, and the medium was changed to exosome depleted DMEM/F-12 conditioned medium containing 10% exosome-free FBS (Xiaopeng, Shanghai, China), 1% antibiotics (penicillin and streptomycin solution) and cultured for 48 h. The culture’s conditioned medium was collected and centrifuged at 300 *g* for 10 min, followed by 2,000 *g* for 10 min to clear the dead cells and cell debris. The supernatant was then centrifuged at 10,000 *g* for 30 min to remove the microcellular vesicles (MVs). Finally, the supernatant was subjected to a 100,000 *g* centrifugation for 70 min to pellet the exosomes and contaminated proteins, the supernatant was discarded and the pellet was washed in cold PBS and ultracentrifuged again at 100,000 *g* centrifugation for 70 min. The supernatant was carefully removed, and the pellet was re-suspended in 100 μl sterile PBS, aliquoted, and stored at −80°C. All centrifugation steps were performed at 4°C.

In each EVs preparation, the concentration of total proteins was quantified by BCA protein assay (Beyotime, China). EVs were characterized by TEM and Western blot analysis to detect the EVs markers, Alix (#2171; Cell Signaling Technology, Danvers, MA, USA), TSG101 (ab83; Abcam) and CD63 (CBL553, Sigma–Aldrich), while calnexin (ab10286, Abcam) as a negative marker was also measured. NTA analysis was also used to measure the concentration and size distribution of EVs.

### Labeling EVs with PKH26

Purified EVs were labeled with a PKH26 kit (red; Sigma–Aldrich) for labeling and uptake studies according to the manufacture’s protocols with some modifications (Zhang et al., 2018). In brief, 250 μl of purified OECs-EVs diluted in PBS mixed with 250 μl of Diluent C. In parallel, 2 μl of PKH26 dye was added to 500 μl of Diluent C for a final PKH26 concentration of 1 × 10^−6^ M, then the mixture was incubated with EVs solution above for 4–6 min at room temperature. Excess dye from the unlabeled EVs was neutralized with 1 ml of 5% BSA and removed by ultracentrifugation. After incubation, the EVs were resuspended in sterile PBS and centrifuged at 100,000 *g* for 70 min twice. The acquired PKH26-labeled EVs pellet was carefully resuspended in 100 μl of PBS. A mixture without EVs was used as a negative control to examine any carryover of PKH26 dye. For negative control, labeling was performed as described but without EVs. PKH26-labeled OECs-EVs were added to DRG cultures in FBS-free medium overnight. The DRG neurons were stained for β-tubulin III (1:200, Abcam; green) and counterstained with DAPI. The internalization of PKH26-labeled OECs-EVs was observed under a fluorescence confocal microscope (A1+, Nikon, Japan).

### Preparation of DRG Explant and Neuron Cultures

DRGs were obtained and purified according protocols described previously (Huang et al., 2018). Briefly, DRGs from neonatal (postnatal day 0–1) rats were dissected and digested with 0.25% typsin (Sigma–Aldrich) for 5 min and then DRG explants were carefully placed on coverslips precoated with rat tail collagen (Invitrogen, Carlsbad, CA, USA). DRGs were maintained in Neurobasal medium (Gibco) supplemented with 2% B27 (Gibco), 50 ng/ml human nerve growth factor (hNGF, R&D Systems, Minneapolis, MN, USA) 2 mM L-glutamine (Gibco) and 1% penicillin-streptomycin. For purification, DRGs were subjected to 10 μM cytosine arabinoside (Sigma–Aldrich) to eliminate contaminated fibroblasts. The medium was replaced with Neurobasal medium (Gibco) without cytosine arabinoside. Subsequently, PBS (control group) and different concentrations of OECs-EVs (10^7^ particles/ml, 10^8^ particles/ml and 10^9^ particles/ml) were added to DRG explants cultures on a daily basis for 3 days. Immunocytochemistry using β-tubulin III (1:200, Abcam) was performed and the axonal length from the edge of DRG explants to the tip of axons as well as the area of neurites and the explant body were measured by Image-Pro Plus 6.0 (Media Cybernetics, Bethesda, MD, USA) software.

DRGs were obtained as described above and were dissected and digested with 0.25% typsin (Sigma–Aldrich) at 37°C for 1 h. DRG neurons were mechanically triturated with a Pasteur pipette and cells were filtered by a 70-μm cell strainer (Thermo Fisher Scientific, Waltham, MA, USA). Cells were seeded at a concentration of 10^5^ cells/ml and maintained at 37°C with 5% CO_2_ in DMEM containing 15% FBS (Gibco) and antibiotics (penicillin and streptomycin solution) for 4 h. The culture medium was replaced with Neurobasal medium (Gibco) supplemented with 2% B27 (Gibco), 50 ng/ml hNGF (R&D systems) 2 mM L-glutamine (Gibco) and 1% penicillin-streptomycin. For purification, DRGs were subjected to 10 μM cytosine arabinoside (Sigma–Aldrich) to eliminate contaminated fibroblasts. The medium was replaced with Neurobasal medium (Gibco) without cytosine arabinoside, PBS (control group) and different concentrations of OECs-EVs (10^7^ particles/ml, 10^8^ particles/ml and 10^9^ particles/ml) were added to DRG neuron cultures on a daily basis for 3 days. Immunocytochemistry using β-tubulin III (1:200, Abcam) and NeuN (1:500, Abcam) was performed and the length of 40 longest axons per condition was measured with Image-Pro Plus 6.0 (Media Cybernetics, Bethesda, MD, USA) software.

Neurite outgrowth was evaluated based on the total area of neurites divided the total area of the explant body and the average length of 40 longest axons as previously described (Zhang et al., 2009).

### Immunofluorescent Staining

Immunofluorescence was performed as previously described (Liu et al., 2017). In brief, cell cultures were fixed with 4% paraformaldehyde for 15 min, and then three 5 min washes in PBS followed by 15 min in 0.1% Triton X-100 (Sigma–Aldrich) for permeabilization and blocked 1 h in 10% normal goat serum at room temperature. Cultures were incubated with primary antibodies overnight at 4°C, three times washed in PBS for 5 min, and incubated with fluorescent conjugated secondary antibodies for 1 h at 37°C. DRG explants were immunofluorescent stained for primary monoclonal mouse anti-β-tubulin III (1:200, Abcam) and reacted with goat anti-mouse Alexa 488 (1:500, Invitrogen, Carlsbad, CA, USA). DRG neurons were immunofluorescent stained for monoclonal mouse anti-β-tubulin III (1:200, Abcam) and polyclonal rabbit anti-NeuN (1:500, Abcam), followed by reaction with goat anti-mouse Alexa 405 (1:500, Invitrogen) and goat anti-rabbit Alexa 488 (1:500, Invitrogen).

### Fabrication of Nerve Conduit

Polycaprolactone (PCL) nerve conduit was prepared following the procedures as described previously (Wang et al., 2015). In brief, 15% (w/v) PCL solution was prepared by dissolving the PCL pellets (Mn = 80,000; Sigma–Aldrich) in a mixture of methanol and chloroform (1:5 v/v; Sigma–Aldrich). PCL solution was loaded in a 10 ml syringe and delivered through a 21-gauge blunt tip syringe needle at a flow rate of 4.5 ml/h by a syringe pump (Cole-Parmer, Vernon Hills, IL, USA). Twelve kilovolt voltage was applied to the electrospun system and the distance between collector and syringe tip was 18 cm. The collector was a 10 cm long and 1.5 cm in diameter rotating stainless steel rod. The PCL nerve conduit was fabricated on the steel rod and dried in vacuum to eliminate the residual PCL solvent at room temperature for 24 h.

### Surgical Procedures

All animal experimental procedures were performed in accordance with the Guide for the Care and Use of Laboratory Animals (National Institutes of Health Publication No 80-23, revised 1996) and approved by the Animal Research Committee of The Fourth Military Medical University, People’s Republic of China. In all experiments, adult SD male rat weighing 220–250 g (purchased from the Laboratory Animal Center of the Fourth Military Medical University) were maintained in a suitable air-filtered environment and fed normally. Attempts were made to reduce the number of animals used and their suffering. All surgical procedures were performed between 9:00 am and 5:00 pm.

The rats were randomly divided into three groups: the autologous graft (autograft) group, the EVs+conduit group, and the PBS+conduit group. Animals were anesthetized intraperitoneally with pentobarbital sodium (1%, 40 mg/kg animal weight). The left sciatic nerve was exposed and created a 5-mm nerve defect. Proximal and distal ends of nerve stumps were both inserted 1 mm into 7 mm long nerve conduit and sutured by three perineural 10/0 nylon sutures. The autograft group, the 5-mm transected nerve segment was sutured in place under the microscope. In the other two groups, the sciatic nerve defect was bridged with a conduit filled with different content. In the EVs+conduit group, the conduit was filled with an OECs-EVs-matrigel mixture (10^8^ particles/ml). For the PBS+conduit group, the conduit was filled with matrigel and PBS which volume is equal to the OECs-EVs.

### Immunofluorescent Staining Assay of Regenerated Nerve

At 4 and 8 weeks after surgery, longitudinal and cross-sections of the regenerated nerves were cut on a paraffin section system. The sections were incubated with mouse anti-β-tubulin III (1:200, Abcam, Cambridge, UK) at 4°C overnight. Next, the primary antibodies reacted with goat anti-mouse IgG FITC (1:200; Abcam, Cambridge, UK) secondary antibodies for 1 h at 37°C. Then the sections were rinsed and observed under a fluorescence microscope (DM6000; Leica, Wetzlar, Germany).

### Morphometric Analysis of Regenerated Axons

At 4 and 8 weeks after surgery, the regenerated nerve segments were harvested and quickly immersed with 3 wt % glutaraldehyde. 1% osmium tetroxide in 0.1 M sodium cacodylate buffer (pH 7.3) was used for further fixation; the nerve segments were dehydrated and embedded in epoxy resin. Transverse semithin (thickness: 1 μm) and ultrathin (thickness: 50 nm) sections were cut from the distal portion of the regenerated nerves. The semithin sections were stained with 1% toluidine blue/1% borax dye solution prepared in deionized water and observed under a light microscope (AH3; Olympus, Japan). While ultrathin sections were stained with uranyl acetate and lead citrate and were examined under a TEM (TECNAI Spirit, FEI, USA). Six semithin and six ultrathin sections were randomly selected from each group of regenerated nerves to quantitatively analyze the axons regeneration and at least six scope fields in each nerve semithin and ultra-semithin section were analyzed according to the sampling scheme method described previously (Mayhew and Sharma, 1984). Axonal regeneration was estimated by the average number of myelinated axons and the average diameter of myelinated axons in the distal portion of the regenerated nerve; the degree of myelination was estimated by the thickness of the myelin sheath and the mean axon to fiber diameter ratio (G-ratio).

### Functional Assays

The motor functional recovery analysis. At 2, 4 and 8 weeks after surgery, functional recovery of the left hind limb of each group were examined by walking track analysis. In brief, the rats were pre-trained to walk across a 10 × 100 cm walking track before surgery. Postoperatively, the rats’ hind paws were immersed with red dye before walking in the track and six paw prints were collected for each animal. The paw length (PL) is the distance between the heel and the third toe, the toe spread (TS) is the distance between the first and the fifth toe and the intermediary toe spread (IT) is the distance between the second and the fourth toe. These measures were all obtained both from the normal (N) and the experimental (E) hind paws. The sciatic function index (SFI) was calculated as formula: SFI = [−38.3 × (EPL − NPL)/NPL] + [109.5 × (ETS − NTS)/NTS] + [13.3 × (EIT − NIT)/NIT] − 8.8. The value of SFI oscillates 0 represents better recovery, while an SFI value of −100 reflects total dysfunction.

Histological analysis of target muscles. At 8 weeks after surgery, the gastrocnemius muscles of the operated hind limb were harvested and weighted. Muscles’ weight was normalized by the normal side to eliminate individual differences of animals. Muscles were cut into transverse sections (thickness of 10 μm) and subjected to Masson trichrome staining. Then the cross-sectional area of muscle fibers was taken from six random fields of sections on the microscope for each group and analyzed with Image-Pro Plus 6.0 (Media Cybernetics, Bethesda, MD, USA) software.

Electrophysiological assessment. At 4 and 8 weeks after surgery, the electrophysiological assessment was performed following the procedures described previously (Huelsenbeck et al., 2012). In brief, animals were anesthetized by isoflurane inhalation and the sciatic nerves were exposed. A bipolar stimulating electrode was placed to contact with the proximal end of the nerve graft and the shaved skin 2 cm from the stimulating cathode respectively. Compound muscle action potentials (CMAPs) from gastrocnemius belly at the ipsilateral side were recorded by a multichannel electrophysiological recorder. For quantitative analysis, the peak amplitude of CMAP, nerve conduction velocity and the latency of CMAP onset was calculated and compared among groups.

### Statistical Analysis

All data were expressed as mean ± SEM. Statistics were calculated using GraphPad Prism (Version 8.0, San Diego, CA, USA). Statistical comparisons of data were analyzed with a one-way analysis of variance (ANOVA) test. Tukey’s *post hoc* test was used to examine the significance of results. Values of *p* < 0.05 were considered statistically significant.

## Results

### Characterization of OECs

**Figure 1:**
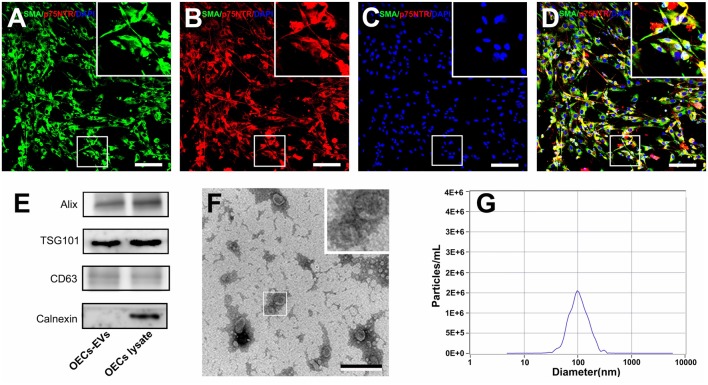
Characterization of olfactory ensheathing cells and isolated EVs. Notes: double immunofluorescent staining of cultured OECs showed the expression of SMA (green; **A**) and p75^NTR^ (red; **B**) with DAPI nuclear counterstaining (blue; **C**). Merge images revealed an OECs purity of more than 96% **(D)**. Western blot analysis of EVs isolated from the supernatant of cultured OECs confirmed the presence of EVs marker proteins of Alix, TSG101, CD63, and the absence of non-exosomal protein (calnexin; **E**). The TEM analysis of isolated EVs **(F)**. Representative traces from nanoparticle tracking analysis (NTA; **G**). The upper right corner of the images **(A–D,F)** is a higher magnification of the boxed area in **(A–D,F)**. Scale bars: **(A–D)** 100μm; **(F)** 200 nm. Abbreviations: OECs, olfactory ensheathing cells; SMA, smooth muscle α-actin; DAPI, 4′,6-diamidino-2-phenylindole; EVs, extracellular vesicles; TEM, transmission electron microscopy; NTA, nanoparticle tracking analysis.

OECs were harvested and purified from SD rat olfactory bulbs and the cell purity was determined by the average ratio of SMA (green) and p75^NTR^ (red) double-positive cell number to DAPI positive cell number. In the present study, the purity of OECs was more than 96% and the representative immunohistochemistry images were shown in Figures 1A–D.

### Characterization of OECs-Derived EVs

We isolated EVs from OECs cultures following standardized methods for EVs isolation. Western blotting confirmed that the isolated preparations carried the characteristic EVs markers Alix, TSG101 and CD63, while calnexin, an integral protein were not detected in EVs (Figure 1E). Moreover, TEM analysis showed the presence of vesicles resembling exosomes in the preparations (Figure 1F). Furthermore, NTA was applied to characterize the vesicles isolated from the OECs cultures and showed a modal peak size of 100.1 ± 26.6 nm indicating that these vesicles were consistent with EVs characteristics (Figure 1G).

### Internalization of OECs-Derived EVs by DRG Neurons

**Figure 2:**
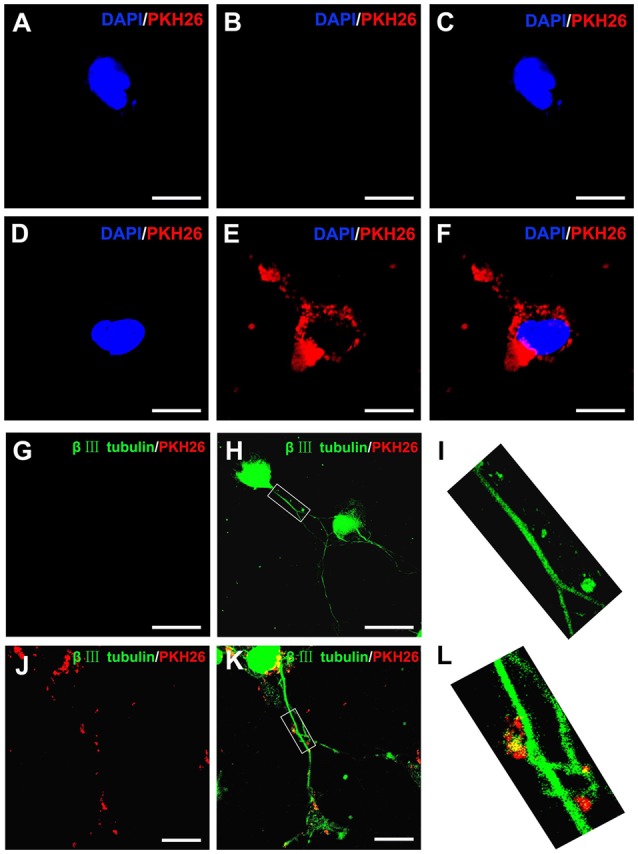
Internalization of OECs-derived EVs by DRG neurons and axons. Notes: the obtained OECs-EVs were labeled by PKH26 (red) and DRG neurons were immunofluorescent stained for β-tubulin III (green) with DAPI nuclear counterstaining (blue). Representative images showed no EVs in the cytoplasm and axons of DRG neurons **(A–C,G–I)** in the control groups, while labeled EVs (red) can be found within the cytoplasm and axons of DRG neurons in the OECs-EVs treated groups **(B–F,J–L)**. Scale bars: **(A–F)** 10 μm; **(G,H)** 50 μm; **(J,K)** 25 μm. Abbreviations: OECs, olfactory ensheathing cells; EVs, extracellular vesicles; DRG, dorsal root ganglion; GFAP, glial fibrillary acidic protein; DAPI, 4′,6-diamidino-2-phenylindole.

It has been reported that upon release, EVs interacted with recipient cells by surface contact, and were internalized by endocytosis or fusion with the plasma membrane of recipient cells (Record et al., 2011). In the present study, PKH26 was added to label OECs-EVs and the labeled OECs-EVs were cocultured with DRG neurons. The PKH26-labeled OECs-EVs were detected in distal axons and cell bodies of DRG neurons, while none was seen in the negative control group, suggesting that OECs-EVs were internalized by DRG neurons (Figures 2A–L).

### OECs-Derived EVs Enhance Axonal Growth *in vitro*

**Figure 3:**
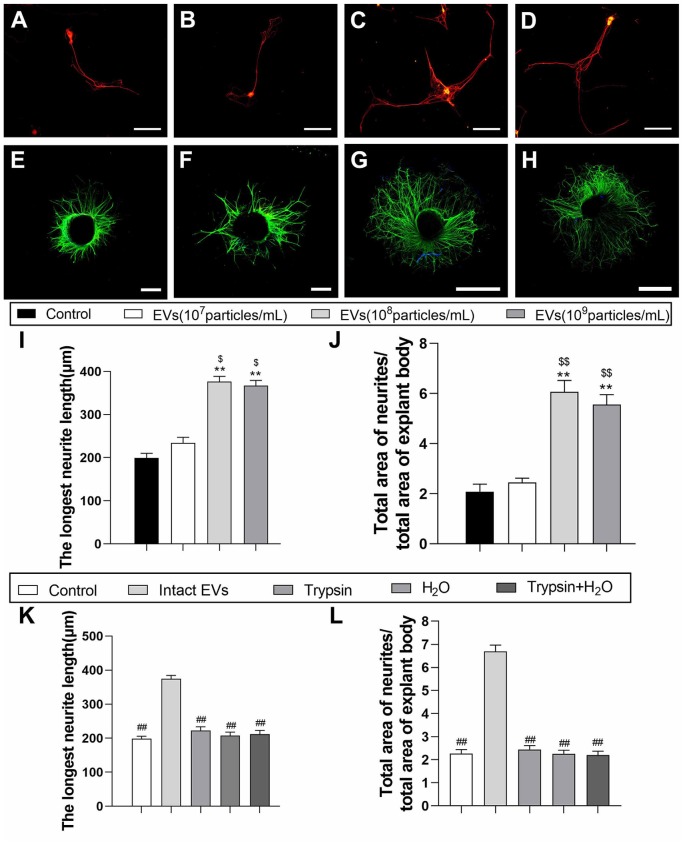
EVs derived from OECs can enhance axonal elongation of DRG neurons and explants. Notes: Representative images of DRG neurons stained for β-tubulin III (red) and NeuN (green), the DRG explants were stained for β-tubulin III (green) with nuclear DAPI counterstaining. Axonal elongation of DRGs after 3 days PBS **(A,E)**, OECs-EVs (10^7^ particles/ml; **B,F**), OECs-EVs (10^8^ particles/ml; **C,G**) and OECs-EVs (10^9^ particles/ml; **D,H**) daily treatment. Treatment of OECs-EVs with different concentrations increased the average length of the longest neurite and the ratio of total area of neurites/total area of explant body **(I,J)**. Effect of pre-treated OECs-EVs on axonal elongation with 3 days daily treatment **(K,L)**. OECs-EVs were incubated with trypsin, lysed by water or treated with trypsin followed by water lysis, control represents PBS treatment; *n* = 4 per group; scale bars: **(A–D)** 100 μm; **(E,F)** 300 μm; **(G,H)** 1,000 μm. The results are expressed as the mean ± SEM. One-way analysis of variance (ANOVA) test with Tukey’s *post hoc* test was used to examine the significance of results. **p < 0.01 for comparison with control group, ^$^*p* < 0.05 and ^$$^*p* < 0.01 for comparison with OECs-EVs (10^7^ particles/ml) group, ^##^*p* < 0.01 for comparison with intact EVs group. Abbreviations: EVs, extracellular vesicles; OECs, olfactory ensheathing cells; DRG, dorsal root ganglion; DAPI, 4′,6-diamidino-2-phenylindole.

To examine the effect of OECs-EVs on neurite outgrowth *in vitro*, the effects of OECs-EVs on purified DRG neurons and DRG explant were studied respectively (Lopez-Leal and Court, 2016; Ren et al., 2019). DRG axon growth rate started to emerge significant difference between OECs-EVs group and PBS control group 3 days after supplementation for the culture system; we selected the time point of 3 days after supplementation to examine in the remaining experiment. First, we evaluated the effects of OECs-EVs on DRG neurons. We found that OECs-EVs applied to DRG neurons significantly increased length of distal axons. Furthermore, we found that the enhancement for neurite outgrowth was dependent on the dosage of OECs-EVs (Figures 3A–D). The longest neutrite length of OECs-EVs (10^8^ particles/ml) treated group (376.3 ± 43.7 μm) was in the similar range to that in the OECs-EVs (10^9^ particles/ml) treated group (366.7 ± 37.6 μm; Figure 3I), which was significantly longer than the PBS control group (199.3 ± 37.3 μm) and OECs-EVs (10^7^ particles/ml) treated group (234.7 ± 41.4 μm; Figure 3I). Second, we evaluated the effects of OECs-EVs on DRG explants (Figures 3E–H). The ratio of total area of neutrites and total area of explant body in OECs-EVs (10^8^ particles/ml) treated group (6.063 ± 0.57) was in the similar range to that in the OECs-EVs (10^9^ particles/ml) treated group (5.557 ± 0.61; Figure 3J), which was significantly higher than that in the PBS control group (2.083 ± 0.38) and OECs-EVs (10^7^ particles/ml) treated group (2.447 ± 0.31; Figure 3J). These data indicated that OECs-EVs stimulate neurite outgrowth *in vitro* and this enhancement on axonal growth was observed only when DRG cultures were treated with EVs with concentration higher than 10^8^ particles/ml (Figures 3I,J). In addition, the OECs-EVs were pretreated with trypsin or distilled water to digest the extracellular proteins or lyse EVs before supplementation, the enhancement of axonal growth in intact OECs-EVs was abolished in OECs-EVs pretreated with trypsin, water or trypsin+H_2_O (Figures 3K,L), suggesting that the beneficial effect of OECs-EVs on neurite outgrowth *in vitro* was dependent on their integrity.

### OECs-Derived EVs Enhance Axonal Regeneration *in vivo*

**Figure 4:**
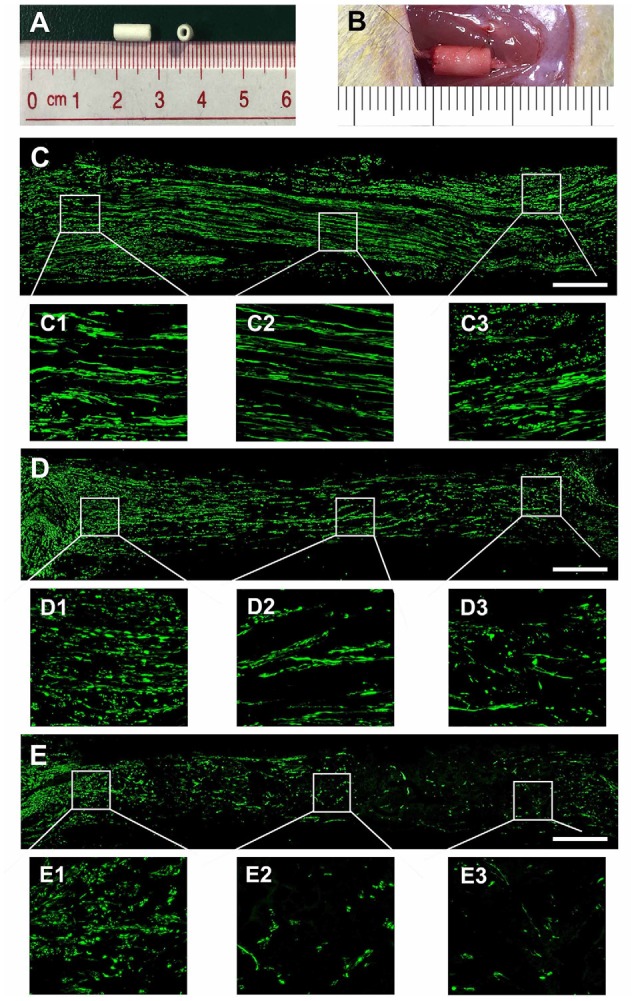
Nerve regeneration in the grafts at 4 weeks. Notes: **(A)** gross appearance of the graft conduit. **(B)** Sciatic nerve graft conduit after implantation. Regenerated nerves were stained for NF160 in the autograft group **(C)**, EVs+conduit group **(D)** and PBS+conduit group **(E)**. The representative images of proximal, middle and distal section of the graft in the autograft **(C1–C3)**, EVs+conduit group **(D1–D3)** and PBS+conduit group **(E1–E3)**; in the EVs+conduit group and PBS+conduit group, in addition to the same volume of EVs and PBS, the same volume of Matrigel^®^ was added to provide a supportive environment; *n* = 4 per group, scale bars: **(C–E)** 500 μm. Abbreviations: EVs, extracellular vesicles.

**Figure 5:**
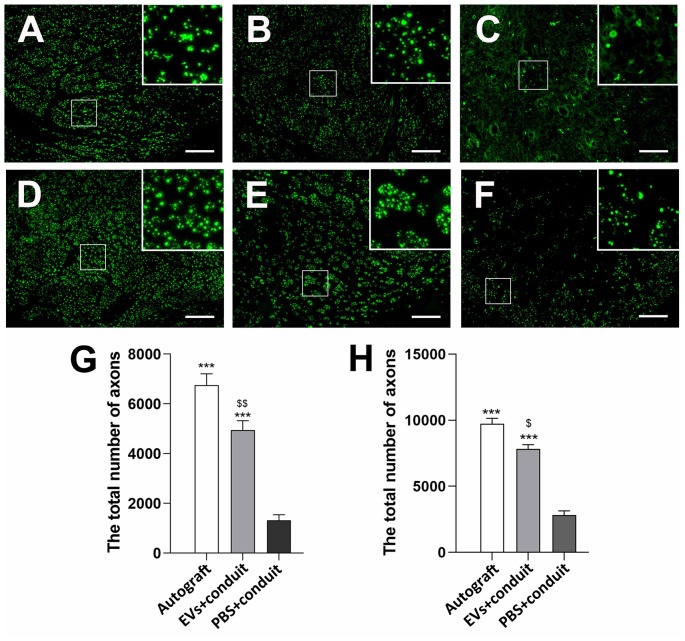
Cross-sections of the distal regenerated nerve segments at 4 and 8 weeks. Notes: representative images of regenerated axons at the distal nerve segment stained for NF-160 (green) in the groups of autograft **(A)**, EVs+conduit **(B)** and PBS+conduit **(C)** at 4 weeks and autograft **(D)**, EVs+conduit **(E)** and PBS+conduit **(F)** at 8 weeks. The upper right corner of the images **(A–F)** is higher magnifications of the boxed area in **(A–F)**. Quantifications of the total number of the regenerated axons in the cross-sections at 4 weeks **(G)** and 8 weeks **(H)**; *n* = 4 per group, scale bars: **(A–F)** 100 μm. The results are expressed as the mean ± SEM. One-way analysis of variance (ANOVA) test with Tukey’s *post hoc* test was used to examine the significance of results. ***p < 0.005 for comparison with the PBS+conduit, ^$^*p* < 0.05 and ^$$^*p* < 0.01 for comparison with the autograft group. Abbreviations: EVs, extracellular vesicles.

To evaluate the effect of OECs-EVs on peripheral nerve regeneration, we created a 5-mm sciatic nerve defect in rats, which was bridged with an autograft or a PCL conduit containing OECs-EVs-matrigel (10^8^ particles/ml) or PBS-matrigel mixture (Figures 4A,B). Axonal regeneration was evaluated at 4 and 8 weeks after surgery by immunofluorescence staining with NF160. At 4 weeks after surgery, NF160 immunostaining showed that the regenerated axons in autograft group (Figure 4C) and the EVs+conduit group (Figure 4D) were evenly distributed from proximal to distal of the graft segments (Figures 4C1–3,D1–3); while the regenerated axons were rarely distributed in the middle and distal of the conduit in the PBS+conduit group (Figures 4E,E1–3). Furthermore, we investigated the total number of NF160 positive axons in the cross-sections of the distal nerve segments at 4 and 8 weeks postoperatively (Figures 5A–F). At 4 week after surgery (Figures 5A–C), NF160 immunostaining showed that few axons were regenerated into the distal segment in the PBS+conduit group (1,321 ± 224), while significantly higher number of axons were found in the autograft (6,751 ± 456) and EVs+conduit groups (4,941 ± 382; *p* < 0.05, Figure 5G). At 8 week after surgery (Figures 5D,E), NF160 immunostaining positive axons in the PBS+conduit group were 2,818 ± 321, while the total number of axons was significantly higher in the autograft (9,730 ± 409) and the EVs+conduit (7,830 ± 377) groups (*p* < 0.05, Figure 5H) in the cross-sections of distal nerve segments.

**Figure 6:**
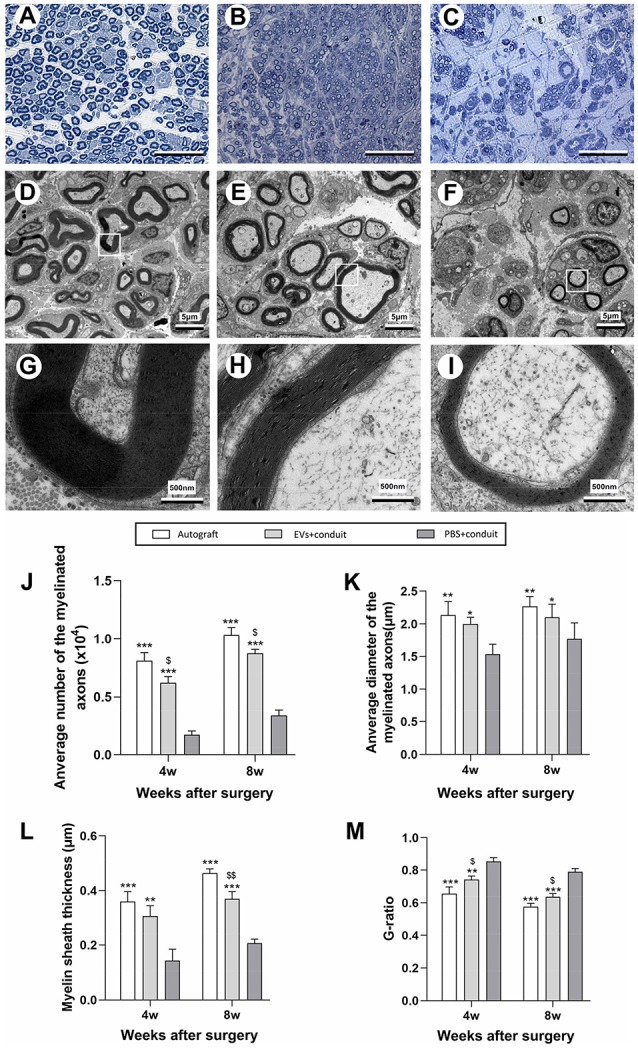
Morphological appearance and morphometric assessments of regenerated nerves in each group. Notes: representative images of toluidine blue staining of regenerated axons in the autograft **(A)**, EVs+conduit **(B)** and PBS+conduit **(C)** in the distal part of the graft of each group at 8 weeks after surgery, respectively. Representative TEM micrographs of regenerated axons **(D–F)** and myelin sheath **(G–I)** in the distal part of the graft in the autograft group **(D,G)**, EVs+conduit group **(E,H)** and PBS+conduit group **(F,H)** at 8 weeks after surgery, respectively. Quantification of the average number of myelinated axons **(J)**, average diameter of myelinated axons **(K)**, myelin sheath thickness **(L)** and G-ratio **(M)**; *n* = 6 per group, scale bars: **(A–C)** 50 μm; **(D–F)** 5 μm; **(G–I)** 5 nm. The results are expressed as the mean ± SEM. One-way analysis of variance (ANOVA) test with Tukey’s *post hoc* test was used to examine the significance of results. *p < 0.05, **p < 0.01 and ***p < 0.005 for comparison with Conduit group, ^$^*p* < 0.05 and ^$$^*p* < 0.01 for comparison with autograft group. Abbreviations: EVs, extracellular vesicles; TEM, transmission electron microscopy.

Morphometric analysis was also performed to investigate nerve regeneration and the degree of myelination of the regenerated axons in each group. The representative images of toluidine blue staining and TEM of regenerated axons were shown in Figures 6A–I. Compared to the PBS+conduit group, more axons (including myelinated and unmyelinated) were found in the autograft (Figures 5A,D,G) and the EVs+conduit groups (Figures 6B,E,H). The morphological appearances of regenerated axons in the autograft and the EVs+conduit groups were also superior to those in the PBS+conduit group (Figures 6A–I). In addition, the average number of the myelinated axons (*p* < 0.05, Figure 6J) and the average diameter of the myelinated axons (*p* < 0.05, Figure 6K) in the EVs+conduit group were in a similar range to those in the autograft group, which were significantly higher than those in the PBS+conduit group. To evaluate the myelination degree of the regenerated axons, myelin sheath thickness and G-ratio were also analyzed at the predefined time point for each group. The thickness of myelin sheath in the EVs+conduit and the autograft groups was significantly better than that of the PBS+conduit group (*p* < 0.05, Figure 6L). The mean G-ratio in the EVs+conduit group was significantly lower than that in the PBS+conduit group, indicating a better degree of myelination was achieved by OECs-EVs (*p* < 0.05, Figure 6M).

### OECs-Derived EVs Promote Neurologic Functional Recovery

**Figure 7:**
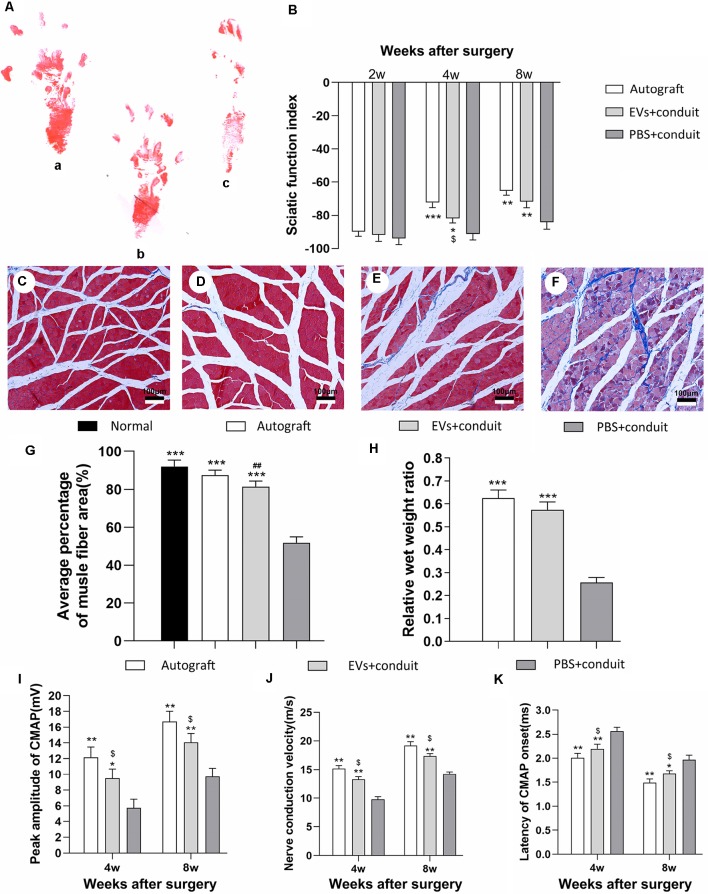
Functional assessment for regenerated nerves. Notes: representative images of operative left footprints **(A)** in the autograft group (a), EVs+conduit group (b) and PBS+conduit group (c) at 8 weeks postoperatively. **(B)** The sciatic function index (SFI) values of each group at 2, 4, and 8 weeks after surgery. Representative light micrographs of gastrocnemius muscles after Masson trichrome staining of the control group (normal muscle; **C)**, the autograft group **(D)**, EVs+conduit group **(E)** and PBS+conduit group **(F)**. **(G)** Quantification of average percentage of muscle fiber area in each group. **(H)** Ratio of injured muscle (ipsilateral) weight to normal muscle (contralateral) weight. The electrophysiological assessment of each group **(I–K)**; the peak amplitude of CMAP **(I)**, the nerve conduction velocity **(J)** and latency of CMAP onset **(K)**; *n* = 6 per group, scale bars: **(C–F)** 100 μm. All data are expressed as the mean ± SEM. One-way analysis of variance (ANOVA) test with Tukey’s *post hoc* test was used to examine the significance of results. *p < 0.05, **p < 0.01 and ***p < 0.005 for comparison with the PBS+conduit group, ^$^*p* < 0.05 for comparison with the autograft group and ^##^*p* < 0.01 for comparison with the control (normal muscle) group. Abbreviations: EVs, extracellular vesicles; CMAP, compound muscle action potential.

Gait analysis is an important index for evaluating the degree of sciatic nerve functions and a gradual return to normal is a sign of successful recovery. Values of SFI were used to evaluate nerve regeneration by measuring rat gait at 2, 4 and 8 weeks after the operation. The representative images of rat footprints for each group were demonstrated in Figure 7A. At 4 and 8 weeks after surgery, the SFI values in the autograft and the EVs+conduit groups were significantly higher than those in the PBS+conduit group (*p* < 0.05, Figure 7B), indicating that the OECs-EVs achieved better motor neurologic functional recovery.

The gastrocnemius is innervated by the sciatic nerve, so the atrophy degree of the gastrocnemius partially reflects the recovery of the sciatic nerve functions (Lavasani et al., 2014). Masson trichrome staining (Figures 7C–F) showed that the average percentage of muscle fiber area in the EVs+conduit group was similar to that in the autograft group, which was significantly higher than that in the PBS+conduit group (*p* < 0.05, Figure 7G). The wet weight of muscle also partially reflects the atrophy degree of the target muscle. The mean weight of gastrocnemius decreased in all groups (excluding the normal muscle) and the relative wet weight ratio of gastrocnemius in EVs+conduit group (0.58 ± 0.08) was in the similar range to that in the autograft group (*p* > 0.05, 0.63 ± 0.09; Figure 7H), which was significantly higher than the PBS+conduit group (*p* < 0.05, 0.28 ± 0.04). These data suggest that the OECs-derived EVs are capable of reducing the degree of target muscle atrophy after nerve injury repair.

Electrophysiological assessments were also performed to further investigate the functional recovery of the repaired nerve at 4 and 8 weeks after surgery (Figures 7I–K). The CMAP is the evoked responses recorded from the target muscle and the latency of CMAP is defined as the lag time between stimulation artifact and the onset of the first deflection of the CMAP curve. Following these assessments, we found that the EVs+conduit group and the autograft group achieved higher peak amplitude of CMAP, faster nerve conduction velocity and shorter latency of CMAP than those in the PBS+conduit group (*p* < 0.05, Figures 7I–K). These data indicate that the OECs-derived EVs are capable of achieving better motor functional recovery in rats with 5-mm sciatic nerve defects.

## Discussion

OECs have been recognized as seeding cells and transplanted into several models of peripheral nerve injuries. OECs share many properties with SCs, but normally do not form myelin in the olfactory system. In spite of this, transplantation of OECs into the sciatic nerve injury lesions showed remyelination of regenerated axons by the transplanted OECs (Dombrowski et al., 2006), which reinforced the idea that the myelin formation property of OECs can be activated when transplanted into an appropriate peripheral pathological environment (Radtke and Kocsis, 2014). It has been reported that the increased secretion levels of growth factors (e.g., NGF, BDNF and neurotrophin-3) from transplanted OECs contribute to their significant effect on enhancing nerve regeneration (Au et al., 2007). Moreover, possible vessel-promoting and anti-inflammatory properties might also be beneficial for nerve regeneration (Kocsis, 2009). Thus, OECs holds many properties that are beneficial for nerve regeneration and functional recovery after nerve injury. Interestingly, many studies have shown that OECs-conditioned medium is capable of enhancing neurite outgrowth (Wang et al., 2003, 2011; Chung et al., 2004). However, the underlying mechanism has been unclear thus far.

Extracellular interaction is an important communication form between cells in the nervous system (Hagiwara et al., 2014; Zhang and Yang, 2018). More and more studies reported that EVs are emerging as a novel and promising information mediator for extracellular interactions (Frühbeis et al., 2013a,b; Janas et al., 2016). It has been shown that EVs are capable of regulating the process of nerve regeneration (Zhang et al., 2017; Ohyashiki et al., 2018; Tkach et al., 2018). EVs derived from glia cells such as astrocytes (Frühbeis et al., 2012), microglia (Potolicchio et al., 2005), and oligodendrocytes (Krämer-Albers et al., 2007) have been demonstrated to serve as modulators of cell-to-cell communication in the healthy and diseased brain. In addition, informative cargoes carried by exosomes, which derived from SCs, MSCs, and macrophages, have already been confirmed to participate in regulating the process of nerve regeneration and enhancing neurite outgrowth (Xin et al., 2013; Pegtel et al., 2014; Zhan et al., 2015; Zhang et al., 2015). However, little information has been obtained about the isolation of EVs derived from OECs and their effect on peripheral nerve repair.

In this study, we first isolated and purified OECs-EVs by serial ultracentrifugation from OECs culture supernatants, and examined the obtained preparations by western blotting, TEM and NTA analysis. Results demonstrated that specific markers of EVs were detected in the preparations, and morphological appearance and size of vesicles were consistent with characteristics of EVs in the preparations. In addition, the obtained OECs-EVs were labeled with PKH26 and the internalization of the labeled EVs by DRG axons and cell bodies were confirmed by immunofluorescent staining assays. Interestingly, we found that OECs-EVs have the capacity in enhancing neurite outgrowth *in vitro*. Furthermore, OECs-EVs also exhibited beneficial effects on nerve regeneration and functional recovery in rats with 5-mm sciatic nerve defects. All these results suggest that the OECs-EVs are capable of promoting nerve regeneration in rats after peripheral nerve injuries. PCL conduit was used in this study to bridge the sciatic nerve gap mainly because of its suitable mechanical strength and toughness. Nerve conduit fillers should be capable of acting as a suitable replacement for fibrin, not prematurely degrade to avoid their scaffolding function is lost, but also must not persist so long as to impede axonal sprouting. Matrigel is heterogeneous and consists primarily of laminin, entactin, collagen, and small concentrations of growth factors such as IGF-1 and TGF-beta, which makes it an excellent surrogate for the native structural matrix and have been shown to induce neurite sprouting of dorsal root ganglia *in vitro* (Pace et al., 2013). Matrigel has been successfully used as nerve conduit filler, which provides a supportive environment for bioactive substances, have been widely used to study the effect of transplanted cells and EVs *in vivo* (Gangadaran et al., 2017). These properties of matrigel are suitable for mixing with EVs as nerve conduit filler to study the effect of EVs *in vivo*.

EVs carry different cargoes, which showed different physiological properties (Eldh et al., 2010). It has been reported that bone marrow- and adipose-MSCs secrete exosomes enriched in distinctive miRNA and tRNA species (Baglio et al., 2015). Interestingly, oligodendrocytes have been found to be capable of secreting exosomes containing major myelin (proteolipid protein and myelin-associated glycoprotein) and stress-protective proteins, which are beneficial for nerve regeneration and remyelination in the CNS (Krämer-Albers et al., 2007). For peripheral nerve regeneration, exosomes derived from SCs have been shown to markedly promote regeneration after sciatic nerve injury (Lopez-Verrilli and Court, 2012; Lopez-Leal and Court, 2016). The Schwann cell-derived exosomes are capable of inducing the growth cone into a pro-regenerative status and decreasing the activity of GTPase RhoA, which participate in axon retraction and cone collapse (Lopez-Verrilli et al., 2013). In addition, many studies have found that various sources of MSCs-derived EVs are capable of promoting nerve regeneration (Lopez-Verrilli et al., 2016), and accumulating evidence suggests that MSCs-derived EVs can deliver their cargo miRNAs to recipient neurons and promote neurite outgrowth (Xin et al., 2012, 2014). In addition, it has also been found that miR-17-92 cluster-enriched exosomes harvested from MSCs are capable of enhancing axonal outgrowth and functional recovery *via* activating PI3K/protein kinase B mechanistic target of rapamycin/glycogen synthase kinase 3β signaling pathway (Xin et al., 2017).

In the previous study, it has been reported that OECs conditioned medium is capable of significantly enhancing neurite outgrowth. In an *in vitro* model which is capable of differentiating the direct and indirect mechanisms underlying the ability of OECs to promote neuronal recovery from injury, it has been found that physical separation using tissue culture inserts (1 μm pore size, permeable to diffusible factors and nanoscale vesicles but not cells) did not completely block the promoting properties of OECs, suggesting that they also secrete soluble factors or nanoscale vesicles which aid neurite outgrowth after nerve injury (Chung et al., 2004). It is important to accurately distinguish OECs from SCs, which is the foundation of this study. OECs and SCs are often confused because OECs and SCs express many of the same phenotypic markers, like p75^NTR^, glial fibrillary acidic protein (GFAP), and S100. It is reported that OECs isolated from the olfactory bulb of embryonic rats (but not SCs isolated from the sciatic nerve of adult rats) express transgelin and calponin, two actin-binding proteins (ABPs) which most often associated with smooth muscle cells. They further found that olfactory glia also express SMA, as well as caldesmon (light isoform) and smooth muscle tropomyosin, two other ABPs. They also demonstrated *in vitro* and *in vivo* that p75^NTR^ positive OECs express SMA, whereas p75^NTR^ positive SCs do not. So they recommend that colocalization of p75^NTR^ and SMA (or calponin) can be used together as definitive markers for OECs both *in vitro* and *in vivo*. In the present study, we firstly isolated EVs from OECs culture medium, and found that OECs-EVs not only enhance neurite outgrowth *in vitro* but also promote nerve regeneration *in vivo*, which might be responsible, at least in part, for the beneficial effect of OECs on peripheral nerve regeneration. In addition, we found that the beneficial effect of OECs-EVs in nerve regeneration is dose-dependent, which is saturated when the dosage of EVs reached 10^9^ particles/ml. It has also been found in the present study that the beneficial effect of OECs-EVs can be abolished by supplementation of trypsin or distilled water, suggesting that the effect of OECs-EVs relies on their integrity. It is a limitation of the present study that the mechanism underlying the beneficial effect of OECs-EVs on nerve regeneration is still unclear. Further studies were needed to identify and screen out the exact components of OECs-EVs in promoting axonal growth by miRNA sequencing or proteomics analysis and verify their effects on nerve regeneration, which might provide new insights for understanding the role of OECs in supporting nerve regeneration, and exploring their potential therapeutic application in nerve injury repair.

## Conclusion

We successfully isolate EVs for the first time from OECs culture medium in the present study. The OECs-EVs are capable of promoting neurite outgrowth *in vitro*, which is dose-dependent and relies on the integrity of the OECs-EVs. In addition, OECs-EVs were used to repair a 5-mm sciatic nerve defect in rats. It has been found that OECs-EVs not only enhance axonal regeneration and myelination of regenerated axons but also promote functional recovery after sciatic nerve injury. These results provide new understandings of OECs in supporting nerve regeneration and highlight their therapeutic potential in nerve injury repair.

## Data Availability Statement

All datasets generated for the present study are included in the article.

## Ethics Statement

All animal experimental procedures in this study were performed in accordance with the Guide for the Care and Use of Laboratory Animals (National Institutes of Health Publication No. 80-23, revised 1996) and approved by the Animal Research Committee of The Fourth Military Medical University, People’s Republic of China.

## Author Contributions

JH and ZL conceived and designed the experiments. BX wrote the manuscript. JG and SL performed the experiments and analyzed the results. LH, TM, LZ, and YY performed the serial ultracentrifugation and analyzed the characterizations of the obtained EVs. All authors revised and approved the final version of the manuscript.

## Conflict of Interest

The authors declare that the research was conducted in the absence of any commercial or financial relationships that could be construed as a potential conflict of interest.
